# Comparison of Two Surgical Approaches for Periacetabular Osteotomy: A Retrospective Study of Patients with Developmental Dysplasia of the Hip

**DOI:** 10.1111/os.14034

**Published:** 2024-03-15

**Authors:** Haitao Guo, Hongfu Jin, Yuanyuan Cheng, Yufeng Mei, Hui Li, Djandan Tadum Arthur Vithran, Shuguang Liu, Jun Li

**Affiliations:** ^1^ Department of Joint Surgery Xi'an Honghui Hospital, Xi'an Jiaotong University Xi'an China; ^2^ Department of Orthopedics Xiangya Hospital of Central South University Changsha China; ^3^ Department of Orthopedics The Second Affiliated Hospital of Air Force Medical University Chongqing China

**Keywords:** Modified Smith–Peterson approach, Modified Stoppa approach, Periacetabular osteotomy

## Abstract

**Objective:**

Given the intricate challenges and potential complications associated with periacetabular osteotomy (PAO) for developmental dysplasia of the hip (DDH). Our study aimed to compare the clinical and imaging benefits and drawbacks of two surgical approaches, the modified Stoppa combined iliac spine approach and the modified Smith–Peterson approach, for treating PAO and to provide guidance for selecting clinical approaches.

**Methods:**

A retrospective analysis of 56 patients with 62 DDHs was conducted from June 2018 to January 2022. The experimental group underwent surgery *via* the modified Stoppa combined iliac spine approach, while the control group underwent surgery *via* the modified Smith–Peterson approach for periacetabular osteotomy and internal fixation. Basic statistical parameters, including age, sex, BMI, and preoperative imaging data, were analyzed. Differences in surgical time, intraoperative blood loss, and postoperative imaging data were compared, as were differences in preoperative and postoperative imaging data between the two groups.

**Results:**

There were 28 hips in the experimental group and 34 in the control group. Moreover, there was no significant difference in the basic parameters between the experimental and control groups. Before and after the operation, for the LCE angle, ACE angle, and Tonnis angle, there was no significant difference in acetabular coverage (*p* > 0.05). However, there were significant differences between the two groups in terms of the above four indicators before and after the operation (*p* < 0.05). After the operation, the experimental group exhibited significant increases in both lateral and anterior acetabular coverage of the femoral head. However, the experimental group had longer operation times and greater bleeding volumes than did the control group. Despite this, the experimental group demonstrated significant advantages in protecting the lateral femoral cutaneous nerve compared to the control group.

**Conclusion:**

The modified Stoppa combined iliac spine approach can be considered a practical approach for PAO and is more suitable for patients with DDH who plan to be treated by one operation than the classic modified Smith–Peterson approach for PAO.

## Graphical abstract

This study analyzed 62 cases of developmental dysplasia of the hip in 56 patients, comparing two surgical approaches for periacetabular osteotomy. The experimental group underwent the modified Stoppa combined iliac spine approach, while the control group received the modified Smith‐Peterson approach. Key findings: (i) both groups had similar preoperative characteristics; (ii) postoperatively, the experimental group exhibited improved acetabular coverage; (iii) the experimental group offered better protection of the lateral femoral cutaneous nerve. However, the experimental group had longer operation times and higher blood loss.

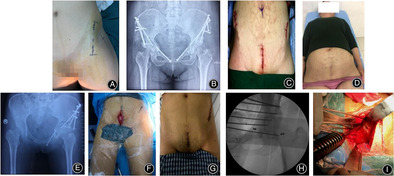

## Introduction

Developmental dysplasia of the hip (DDH) is a common orthopedic ailment characterized by inadequate acetabular coverage of the femoral head, primarily characterized by insufficient anterior and lateral wrapping on imaging.^1^ When left untreated, this procedure can lead to hip impingement, hip arthritis, and femoral head necrosis. Early diagnosis and surgical intervention are crucial, especially for younger patients. Periacetabular osteotomy (PAO), developed by Professor Ganz's team at the University of Bern, Switzerland, is a clinical treatment for developmental hip dysplasia that has gained worldwide recognition and application since its first clinical use in 1988.^2^, ^3^ This procedure moves the hip joint's rotation center inwards, enhancing acetabular coverage, increasing the contact area of the weight‐bearing joint surface, and reducing cartilage stress on the acetabulum and the weight‐bearing surface of the femoral head, thereby preventing and delaying the onset of hip arthritis. However, due to the deep osteotomy site and the wide distribution of blood vessels and nerves around the hip joint, this operation demands a high level of surgical skill from the operator, has a prolonged learning curve, and is susceptible to severe surgical complications.^4^, ^5^ To address these challenges, surgeons are continually exploring novel surgical approaches to increase surgical success rates and minimize surgical complications.^6^

Bernese PAO, as described by Ganz *et al*., is frequently utilized as a reorientation osteotomy in adolescents.^7^, ^8^ This specific surgical procedure is designed to maintain the structural integrity of the posterior column, thereby providing a degree of stability. Nevertheless, a notable drawback of Bernese PAO is the occurrence of joint penetration, which is linked to the difficulty of directly visualizing the quadrilateral surface during the operation. Another consideration is the potential for residual scarring due to the extensive exposure involved in the procedure.^9^ Various incision modifications have been developed in response to extensive muscle dissection, resulting in wide wound scarring associated with the Smith–Peterson incision, which was initially employed in Bernese osteotomy.^10^, ^11^ The Stoppa approach has been regularly implemented in general surgery with reasonable safety, but there have been no reports regarding its application in orthopedic PAO surgery.^12^ Drawing inspiration from the approach's use in periacetabular fractures, our team reviewed the relevant literature and designed an improved Stoppa approach combined with the iliac spine approach for performing periacetabular osteotomy.^13^, ^14^ The purposes of our study were as follows: (i) compare the improved Stoppa approach combined with the iliac spine approach with the classical S‐P approach based on basic statistical parameters and compare the differences in surgical time, intraoperative blood loss, and postoperative imaging data between the two groups; and (ii) analyze the advantages and disadvantages of this modified Stoppa approach combined with the iliac spine approach, providing a reference for selecting better surgical approaches in clinical practice.

## Materials and Methods

### Inclusion and Exclusion Criteria

Ethics approval and consent to participate were obtained for this study in accordance with the principles outlined in the Declaration of Helsinki. Approval was granted by the Ethics Committee of Hong Hui Hospital (IRB: 2018HHH‐CR016). The inclusion criteria were as follows: (i) adult with DDH (Crowe I–II grade 0–I); and (ii) underwent surgical treatment (modified Smith–Peterson approach or modified Stoppa combined with the iliac spine approach). The exclusion criteria were as follows: (i) had surgery around the hip joint or in the abdominal area; (ii) had hip joint incongruency; and (iii) had advanced osteoarthritis (Tönnis grade > II).

Between June 2018 and January 2022, a total of 62 primary dysplastic hips from 56 patients were systematically chosen in our department. This selection comprised eight males (involving 10 hips, with two patients exhibiting bilateral DDH) and 48 females (involving 52 hips, with four patients presenting bilateral DDH). None of the patients had a history of hip or abdominal surgery within the past 6 weeks, and all had a lateral center edge angle (LCE) less than 25° or a Tonnis angle greater than 10°. The surgical interventions included periacetabular osteotomy and orthopedic and internal fixation, with a modified Smith–Peterson approach used in 34 hips (28 single and three double hips) (S‐P Group) and a modified Stoppa combined with the iliac spine approach used in 28 hips (22 single and three double hips) (Stoppa Group).

### Surgical Methods

#### Modified Smith–Peterson Approach

**FIGURE 1:**
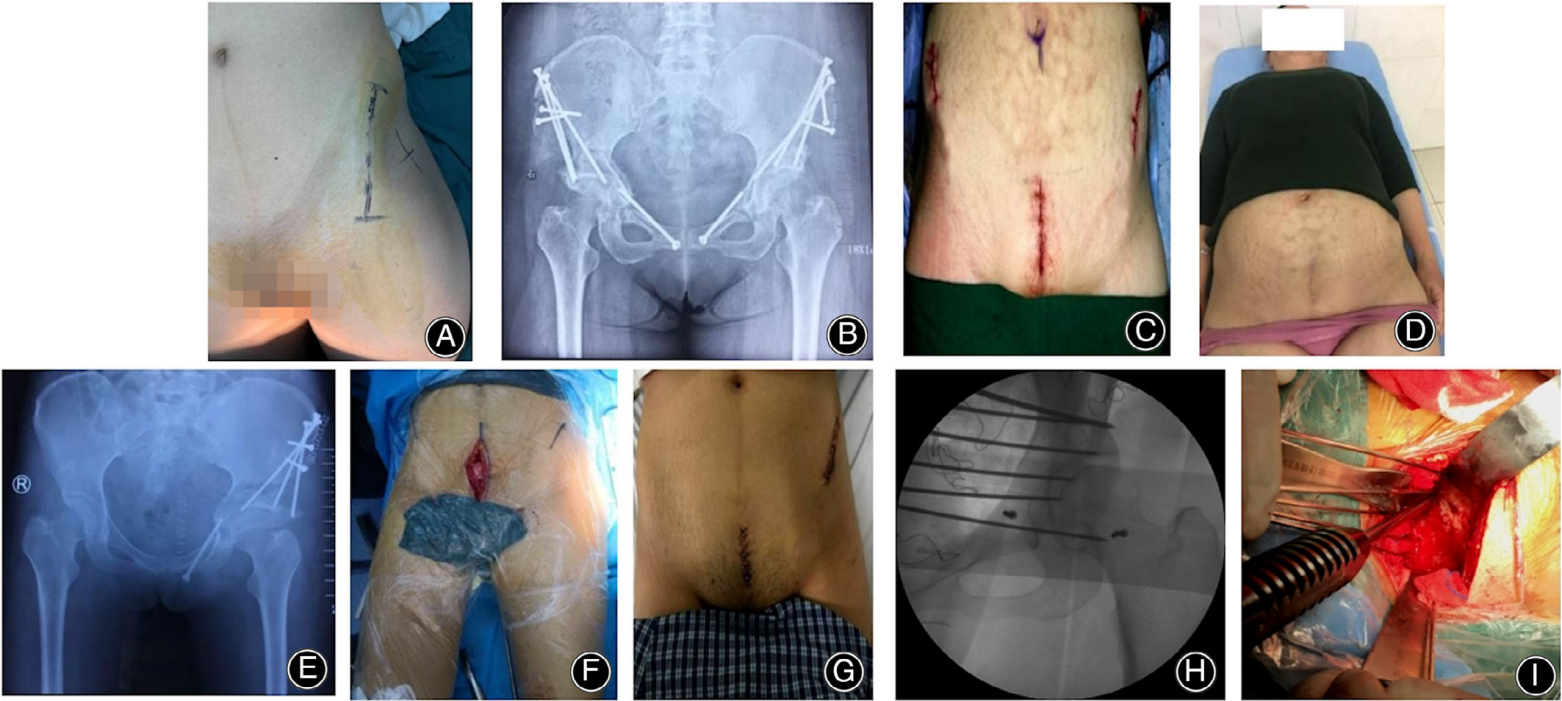
Two examples of patients with developmental dysplasia of the hip. (A) The incision of modified smith‐peterson approach. (B–D) A postoperative pelvic anteroposterior X‐ray and incision appearance of a patient with bilateral hip dysplasia using Stoppa combined with the iliac spine approach; (E–G) A postoperative anteroposterior pelvic radiograph of a patient with unilateral hip dysplasia using Stoppa combined with the iliac spine approach and the intraoperative and postoperative incision appearance. (H, I) The exposed quadrilateral area of the acetabulum during surgery.

A modified S‐P incision ~12–16 cm in length was made from the anterior superior iliac spine forward and down. The skin, and fascia were cut in turn, and a 1 × 1 × 2 cm bone block was used to chip off the anterior superior iliac spine to separate the origin of the sartorius muscle and the inguinal ligament (Figure 1A). The fascia lata and sartorius muscle were removed, and attention was given to protecting the lateral femoral cutaneous nerve. Between the lateral side of the iliopsoas tendon and the medial edge of the femoral neck, a special bone curette was placed against the ischial ramus, and the osteotomy of the ischial ramus was completed by fluoroscopy. The medial side of the iliac plate was dissected subperiosteally, and the straight head and reverse head of the rectus femoris were dissected to expose the inner wall of the iliac plate, the quadrilateral body, and the superior pubic ramus. Further osteotomy of the superior ramus of the pubis, osteotomy of the acetabular roof, and osteotomy of the quadratus were performed.

The osteotomy block was rotated with the proximal suprapubic branch as a hinge and fixed with 3–4 4.5 mm 80–100 mm screws. The incisions were closed layer by layer, and drainage tubes were placed to control bleeding. This surgical technique allows excellent rotation of the osteotomy and good coverage of the hip joint.^15^

#### Modified Stoppa Combined Iliac Spine Approach

**FIGURE 2:**
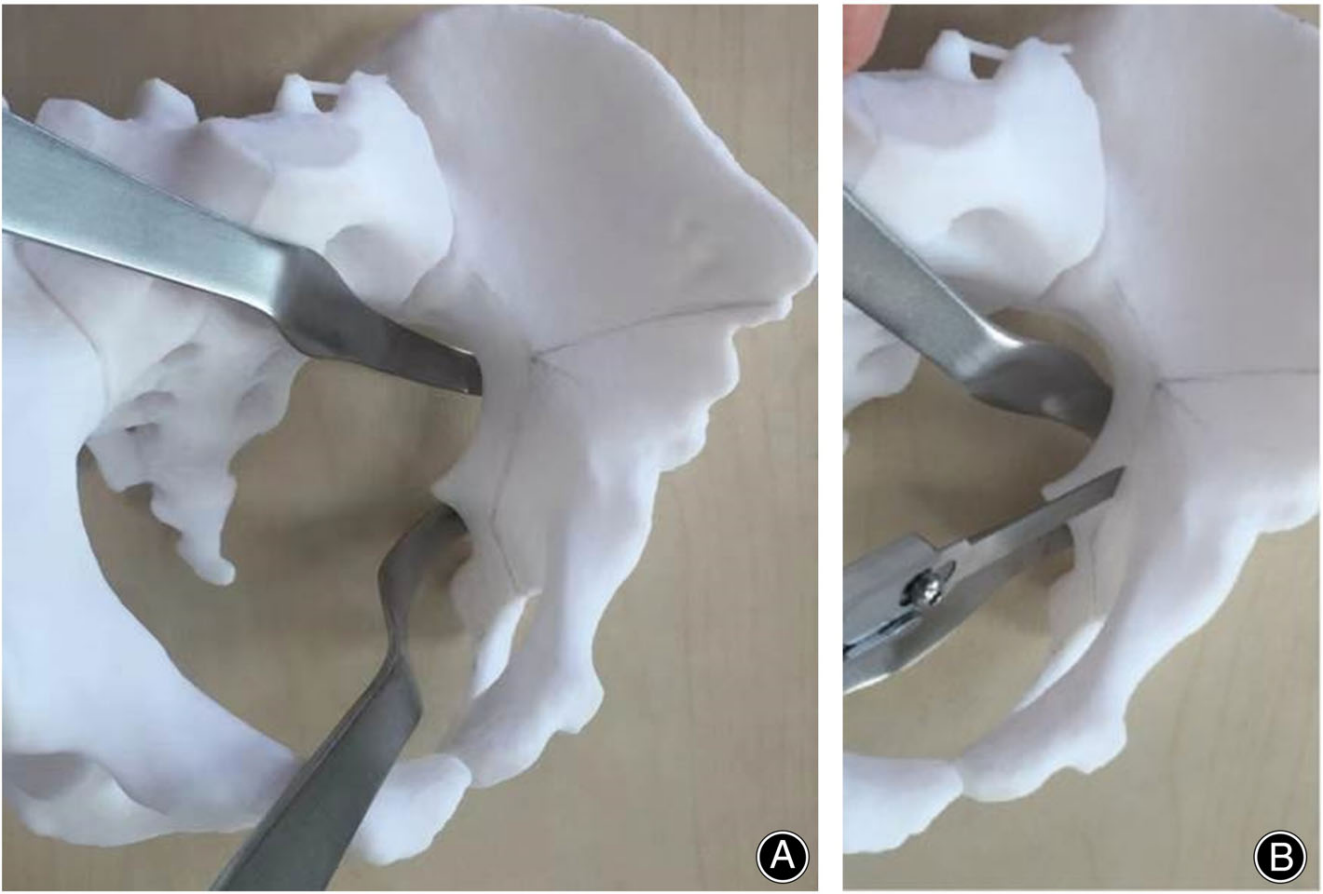
Quadrilateral osteotomy in the 3D‐printed pelvic model. (A) The quadrilateral can be wholly exposed during the operation. (B) Using the modified Stoppa combined iliac spine approach, osteotomy was completed under direct vision.

To perform the surgical procedure, a Stoppa incision 7–8 cm in length was made in the central part of the abdomen (Figure 1B–G). The incision is then made layer by layer through the skin, subcutaneous tissue, and fascia to expose the rectus abdominis sheath. The aponeurosis was incised longitudinally, and the rectus abdominis muscle was separated along the white line to reveal the superior pubic branch and quadrilateral space, along with the extraperitoneal space. During this step, care should be taken not to damage the external iliac vessels or obturator neurovascular bundles but rather to ligate the corona Mortis vessel. The pubic branch was then osteotomized after confirming the direction of the quadrilateral osteotomy using a guide pin. The acetabular bottom was then osteotomized, and ischial osteotomy was performed by separating the starting point of the internal obturator muscle parallel to the lower edge of the acetabulum (Figure 2A,B). The anterior superior iliac spine was approached and osteotomized sequentially, followed by apical acetabular osteotomy in the direction of the ischial notch. The osteotomy block was then rotated using the proximal suprapubic branch as a hinge and temporarily fixed. Fluoroscopy is used to confirm proper rotation of the osteotomy and good coverage of the hip joint. Finally, the osteotomy block was fixed in place with three to four 4.5 mm screws, varying in length from 80 to 100 mm, bleeding was controlled, and the two incisions were closed layer by layer, with drainage tubes placed.^16^ The exposed quadrilateral area of the acetabulum during surgery is shown in Figure 1H,I.

### Observed Indicators

The observation indices for this study included basic clinical parameters, including age, sex, side (left/right), weight, height, and body mass index (BMI), between the two groups before the operation.^17^ The imaging parameters that were compared included the lateral center edge angle (LCE), anterior center edge angle (ACE), Tonnis angle, and acetabular coverage. The parameters that were compared between the two groups included intraoperative blood loss, operation time, incidence of early postoperative complications, and the abovementioned four imaging indices. The imaging indices for each of the four indicators were compared between the two groups postoperatively. Early postoperative complications in this study included poor wound healing, infection, femoral nerve symptoms, lateral femoral cutaneous nerve symptoms, and sciatic nerve stimulation symptoms.^18^

### Statistical Methods

All the statistical analyses were performed using SPSS Statistics 25 (version 25.0.0.0, IBM, Armonk, NY, USA). The primary clinical parameters before the operation, including side and sex (left/right), were analyzed using the *χ* ^2^ test. Age, weight, height, BMI, and preoperative imaging parameters were analyzed using an independent sample *t* test. The volume of blood loss, operation time, incidence of early complications, and postoperative imaging parameters were also examined using an independent sample *t* test. Additionally, paired sample *t* tests were performed for imaging indices before and after the operation within each group. *p* < 0.05 indicated statistical significance.

## Results

### Comparative Analysis of Basic and Clinical Data between the Two Groups

The primary data from 62 hips of 56 patients were registered and analyzed in this study and are compared in Table 1. A total of 34 hips were affected in 31 patients in the S‐P group, and 28 hips were affected in 25 patients in the Stoppa group based on the different incisions. There were no significant differences between the two groups regarding sex, age, weight, height, BMI, or left/right hip joint. However, the Stoppa group had longer operation times and greater bleeding volumes than did the S‐P group (Table 1).

**TABLE 1:** Comparison of primary data of the patients between the two groups.

Parameter	Group Stoppa	Group S‐P	*F*/*χ* ^2^ value	*p* value
Number of patients (hips)	25 (28)	31 (34)	‐	‐
Number of side (right/left)	12/16	16/18	0.109	0.471
Age (years)	25.60 + 6.52	29.20 ± 7.83	0.113	0.405
Sex (male/female)	3/22	5/26	0.193	0.482
Weight (Kg)	59.60 + 3.87	55.00 ± 4.59	0.041	0.760
Height (cm)	164.50 ± 6.54	164.30 ± 7.63	0.469	0.457
BMI (kg/m^2^)	22.07 ± 1.51	20.44 ± 2.07	1.335	0.053
Operation time (minutes)	231.00 ± 42.28	132.50 ± 15.86	1.550	0.000
Blood loss (mL)	973.00 ± 92.11	815.00 ± 163.38	5.668	0.032

### Complications

After the operation, there were two cases of superficial wound infection in the S‐P group and one case in the Stoppa group with wound fat liquefaction. Three patients healed after the dressing change. In the S‐P group, symptoms related to the involvement of the lateral femoral cutaneous nerve were noted in nine patients and were characterized by hypoesthesia in the anterolateral thigh region. Symptoms resolved within 3–6 months postoperatively in six patients, while two patients exhibited persistent symptoms for up to 1 year; in one patient, symptoms persisted for a duration of 2 years following the operation. No hypoesthesia caused by lateral femoral cutaneous nerve injury occurred in the Stoppa group.

### Comparative Analysis of Imaging Data between the Two Groups

Regarding the preoperative imaging data, there was no statistically significant difference between the S‐P group and the Stoppa group in terms of the LCE angle, ACE angle, Tonnis angle, or acetabular coverage (*p* > 0.05) (Table 2). Additionally, no statistically significant differences were observed between the two groups postoperatively in terms of the LCE angle, ACE angle, Tonnis angle, or acetabular coverage (*p* > 0.05) (Table 3). However, when comparing the pre‐ and postoperative data for each group, a statistically significant difference was observed in the four indicators mentioned above (*p* < 0.05) (Tables 4 and 5). Postoperatively, there was a significant increase in lateral and anterior coverage of the acetabulum on the femoral head.

**TABLE 2:** Comparison of preoperative imaging data between the two groups.

Surgical approach	Preoperative LCE °	Preoperative ACE °	Preoperative Tonnis °	Preoperative acetabular coverage (%)
Group S‐P	1.786 ± 7.151	−0.571 ± 8.167	20.357 ± 5.807	59.82 ± 12.21
Group Stoppa	1.400 ± 6.995	−2.500 ± 10.157	21.300 ± 6.378	59.65 ± 10.00
*F*‐value	0.022	0.361	0.185	0.450
*p*‐value	0.884	0.551	0.670	0.510

**TABLE 3:** Comparison of postoperative imaging data between the two groups.

Surgical approach	Postoperative LCE °	Postoperative ACE °	Postoperative Tonnis °	Postoperative acetabular coverage (%)
Group S‐P	23.821 ± 4.884	33.893 ± 6.315	4.464 ± 3.776	82.12 ± 5.91
Group Stoppa	25.200 ± 4.756	34.300 ± 9.322	4.700 ± 5.519	81.83 ± 4.85
*F*‐value	0.595	0.024	0.022	0.226
*p*‐value	0.446	0.879	0.882	0.640

**TABLE 4:** Comparison of preoperative and postoperative imaging data in the S‐P group.

Surgical approach	LCE°	ACE°	Tonnis°	Acetabular coverage (%)
Preoperative	1.786 ± 7.151	−0.571 ± 8.167	20.357 ± 5.807	59.82 ± 12.21
Postoperative	23.821 ± 4.884	33.893 ± 6.315	4.464 ± 3.776	82.12 ± 5.91
*p*‐value	0.000	0.000	0.000	0.000

**TABLE 5:** Comparison of preoperative and postoperative imaging data in the Stoppa group.

Surgical approach	LCE°	ACE°	Tonnis°	Acetabular coverage (%)
Preoperative	1.400 ± 6.995	−2.500 ± 10.157	21.300 ± 6.378	59.65 ± 10.00
Postoperative	25.200 ± 4.756	34.300 ± 9.322	4.700 ± 5.519	81.83 ± 4.85
*p*‐value	0.000	0.000	0.000	0.000

## Discussion

### Summary

In summary, our study compared the clinical and imaging outcomes of two surgical approaches for PAO: the modified Stoppa combined iliac spine approach and the classic modified S‐P approach. These findings suggest that this modified Stoppa approach combined with the iliac spine approach is a practical and preferable option for treating PAO.

The choice of surgical approach for periacetabular osteotomy is evolving as surgeons become more familiar with the technique and consider perioperative complications and patient concerns.^19^ Various approaches, including the iliac groin approach, the Smith–Peterson approach with double incisions, and the modified Smith–Peterson approach, have been used.^20^, ^21^ However, these approaches often result in long incisions that cause significant soft tissue damage, increase the risk of complications, prolong hospital stays, and leave unsightly scars. On the basis of a cadaver study by Elmadağ and Acar, our department explored the feasibility of using the modified Stoppa incision for periacetabular osteotomy.^22^, ^23^ We conducted a simulated osteotomy in cadavers and applied the technique in clinical practice. In this study, we found that the modified Stoppa combined iliac spine approach has advantages over the modified Smith–Peterson approach.

### Comparison of Incisions

A comprehensive comparison of surgical incisions delineates the unique attributes of the modified Smith–Peterson and modified Stoppa techniques. The former is characterized by a lengthy arc‐shaped incision, whereas the latter, when integrated with the iliac spine approach, features two shorter, linear incisions that boast a discreet profile. Importantly, both approaches offer a cosmetic advantage, as the incisions can be discretely concealed beneath women's triangular underwear, catering to the preferences of patients who prioritize aesthetic outcomes (Figure 1D–G).

Moreover, the modified Stoppa incision, positioned along the midline of the abdomen, is particularly important for patients who require bilateral hip surgery simultaneously. This strategic placement enables comprehensive coverage of both the pubic branches and the quadrilateral body, optimizing surgical access and facilitating streamlined operative management.

In addition to its cosmetic and bilateral surgical advantages, the Stoppa approach provides access to critical anatomical regions, including the extraperitoneal pelvic space and the anterior bladder area. Furthermore, the flexibility of extending the incision in requisite directions enhances surgical visualization and maneuverability within the operative field, promoting precision and efficacy in surgical interventions.

### Vascular Injury

**FIGURE 3:**
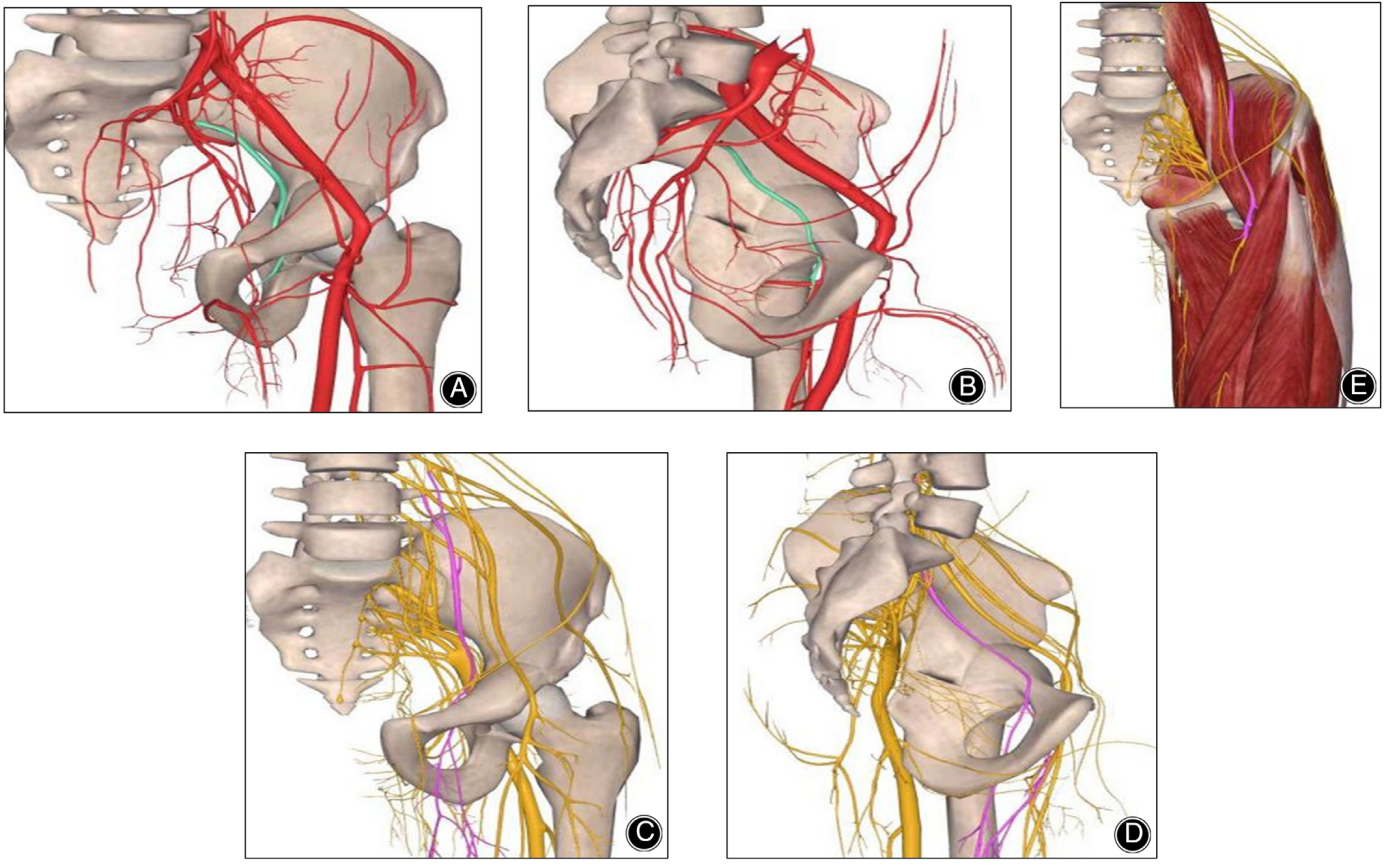
Anatomical location of the obturator vessels, obturator, and femoral nerves (images from 3D body). (A) Movement of the obturator vessel (green) in the anteroposterior position of the pelvis; (B) Movement of the obturator vessel (green) in the medial position of the pelvis; (C) Movement of the obturator nerve (purple) in the anteroposterior position of the pelvis; (D) Movement of the obturator nerve (purple) in the medial position of the pelvis; (E) Movement of the femoral nerve (purple) and surrounding muscles.

Moreover, PAO poses inherent risks of pelvic vessel injury, potentially resulting in significant hemorrhage during acetabular osteotomy procedures. In certain instances of severe bleeding, vascular embolism may be deemed necessary for hemorrhage control.^7^ Notably, Elmadağ *et al*. underscored the substantial risk of vascular injury, highlighting an average distance ranging between 27.2 and 33.4 mm from the superior pubic branch to the obturator vessel^24^ (Figure 3A,B).

However, within the Stoppa group, which adopted an alternative approach to surgical intervention, notable advantages in vascular preservation were observed. Specifically, the obturator vessel, identified as particularly susceptible to injury in traditional methodologies such as the Smith–Peterson approach, benefits from enhanced protection afforded by direct visual access. In both groups, no instances of injury to the obturator vessels were noted. However, within the Stoppa group, we can more confidently proceed with pubic ramus and quadrilateral plate osteotomies, owing to the enhanced visualization of the vascular structures and osteotomy sites.

This heightened intraoperative visibility contributes to effective safeguarding of critical vascular structures, minimizing the likelihood of inadvertent injury during surgical manipulation.

### Nerve Injury

In our study, the comparative assessment of neurological complications between the two cohorts primarily focused on injury to the lateral femoral cutaneous nerve (LFCN). Neither group exhibited involvement of the obturator nerve or the sciatic nerve, while manifestations of sciatic nerve injury were absent. Notably, the S‐P group presented with nine cases of LFCN injury, whereas no instances of LFCN injury were observed in the Stoppa group. Similar to the obturator vessels mentioned previously, the obturator nerve, being amenable to exposure and direct visualization, was adequately safeguarded within the Stoppa group.

In the study conducted by Wall *et al*., a critical observation emerged revealing that during superior pubic branch osteotomy, the obturator nerve was ~4–8 mm from the cutaneous branch. This proximity elevated the susceptibility to obturator nerve injury, particularly when employing the modified Smith–Peterson approach, wherein the nerve remained unexposed (Figure 3C,D). In stark contrast, the modified Stoppa approach demonstrated a distinct advantage, enabling direct visualization and precise protection of the obturator nerve situated adjacent to the superior pubic branch. This nuanced approach proves particularly advantageous for individuals with acetabular double‐column fractures who have experienced preexisting obturator nerve injuries.

Furthermore, the modified Smith–Peterson approach, characterized by the separation of the tensor fascia lata from the rectus femoris, carries the potential risk of inducing femoral nerve paralysis. Additionally, injury to the lateral femoral cutaneous nerve during this approach may result in permanent sensory disturbance of the anterolateral thigh, significantly diminishing postosteotomy patient satisfaction. In contrast, the modified Stoppa approach strategically mitigates the incidence of femoral nerve injury by tactically separating the femoral nerve and cutaneous branch during pubic branch osteotomy. Moreover, the Stoppa approach demonstrated a significantly lower probability of inflicting damage to the lateral femoral cutaneous nerve than did the Smith–Peterson approach. This discrepancy is attributed to the fact that the iliac spine incision in the Stoppa approach generally does not extend 3 cm below the anterior superior iliac spine (Figure 3E).

Consequently, the modified Stoppa approach emerges as a highly recommended choice for patients grappling with acetabular double‐column fractures, offering a pivotal reduction in the risk of nerve injuries during surgical interventions. Al Adawy *et al*. further endorsed this approach, emphasizing its utility in facilitating direct exploration and treatment of obturator nerve injuries encountered during surgery.^25^

### Internal Fixation Method

In both groups, comprising patients from both the Smith–Peterson and Stoppa groups, a standardized fixation protocol was used, involving the use of three to four 4.5 mm screws, varying in length from 80 to 100 mm, for triangular fixation. Notably, in instances where the osteotomy site of the superior pubic branch exhibited significant separation subsequent to rotation, a strategic approach was employed. Specifically, a screw was used to secure the pubic branch outwardly from the pubic symphysis. In contrast, within the Stoppa group, the iliac bone block was affixed using screws.

This meticulous fixation strategy not only streamlines the surgical procedure but also enhances the overall strength of fixation, consequently minimizing postoperative discomfort arising from excessive friction at the fractured end of the pubic branch osteotomy site, which contributes to the optimization of surgical outcomes while concurrently mitigating potential complications associated with inadequate stabilization. Furthermore, this approach underscores the importance of tailored fixation techniques in ensuring optimal postoperative recovery and long‐term patient satisfaction.^26^, ^27^

### Other Complications

One complication of PAO, with an incidence of ~2.7%, is that the osteotome may cut into the joint.^9^ This is mainly due to the need for simultaneous fluoroscopy and osteotomy during the quadrilateral osteotomy procedure. Some scholars have attempted to guide osteotomy by making marks on the quadrilateral. Shazar et al. retrospectively analyzed 122 cases of acetabular fractures and reported that the improved Stoppa approach provided better exposure to the quadrilateral area and was advantageous in terms of reducing quality, operation time, and early postoperative complications.^28^ Hammad and El‐Khadrawe also suggested that the improved Stoppa approach was advantageous, particularly in patients with double‐column fractures combined with quadrilateral fractures, where direct fixation with supporting plates is possible.^29^, ^30^ In the modified Stoppa approach, the quadrilateral and acetabulum bottoms are approached from the inside, close to the acetabulum and quadrilateral. This study did not observe any severe complications associated with this approach.

### Advantages of this Technique

Compared with the modified S‐P incision, the advantages of this approach reported in our study are as follows: (i) the quadrilateral can be fully exposed, and osteotomy can be performed under direct vision; additionally, the risk of injury to the obturator nerve and vessels can be fully reduced; (ii) the incision is more concealed than the modified S‐P incision; (iii) there is almost no risk of injury to the lateral femoral cutaneous nerve; and (iv) the pubic ramus can be fixed laterally with long screws from the symphysis pubis, which is more conducive to healing.

### Strengths and Limitations of this Study

The strength of this study is the use of a PAO surgical approach that can be applied clinically on the basis of the findings of cadaveric studies. The limitation of this study is that because it is a surgical approach for PAO that was initially applied in the clinic, only early complications of this surgical approach were counted, and further cases and follow‐up are needed for long‐term complications and curative effects. Future clinical research should focus on the following key aspects, such as long‐term complication assessment, large‐scale studies, quality of life and functional recovery assessment, to augment our understanding and refine the applicability of this PAO surgical technique.

## Conclusion

The modified Stoppa approach, in combination with the iliac spine approach, can achieve comparable imaging angle correction and good acetabular coverage of the femoral head compared to the widely accepted modified Smith–Peterson approach. Direct visualization *via* the pubic branch, posterior column of the acetabulum, and quadrilateral osteotomy improves the accuracy and safety of osteotomy, reduces the need for intraoperative fluoroscopy, and decreases the incidence of vascular, obturator nerve, femoral nerve, and lateral femoral cutaneous nerve injuries. Postoperative wounds are well conserved, which is advantageous for single placement and a single operation to treat double hip disease. Therefore, the modified Stoppa approach represents a superior option for periacetabular osteotomy in clinical practice.

## Conflict of Interest Statement

The authors declare that the research was conducted without any commercial or financial relationships construed as potential conflicts of interest.

## Ethics Statement

The study was conducted in accordance with the Declaration of Helsinki and approved by the Ethics Committee of Hong Hui Hospital (Approval Notice Number: 2018HHH‐CR016).

## Author Contributions

HTG and HFJ wrote the manuscript, and YYC and YFM prepared the figures. HL and DTAV revised this manuscript. SGL and JL conceptualized this study. All the authors approved the final version of the manuscript and agreed to be accountable for all aspects of the work.
